# Prediction of novel miRNAs and associated target genes in *Glycine max*

**DOI:** 10.1186/1471-2105-11-S1-S14

**Published:** 2010-01-18

**Authors:** Trupti Joshi, Zhe Yan, Marc Libault, Dong-Hoon Jeong, Sunhee Park, Pamela J Green, D Janine Sherrier, Andrew Farmer, Greg May, Blake C Meyers, Dong Xu, Gary Stacey

**Affiliations:** 1 Digital Biology Laboratory, Computer Science Department and Christopher S. Bond Life Sciences Center, University of Missouri, Columbia, MO 65211, USA; 2 Division of Plant Sciences, National Center for Soybean Biotechnology, C.S. Bond Life Sciences Center, University of Missouri, Columbia, MO 65211, USA; 3 Department of Plant and Soil Sciences and Delaware Biotechnology Institute, University of Delaware, Newark, DE 19711, USA; 4 National Center for Genome Resources, 2935 Rodeo Park Drive East, Santa Fe, NM 87505, USA

## Abstract

**Background:**

Small non-coding RNAs (21 to 24 nucleotides) regulate a number of developmental processes in plants and animals by silencing genes using multiple mechanisms. Among these, the most conserved classes are microRNAs (miRNAs) and small interfering RNAs (siRNAs), both of which are produced by RNase III-like enzymes called Dicers. Many plant miRNAs play critical roles in nutrient homeostasis, developmental processes, abiotic stress and pathogen responses. Currently, only 70 miRNA have been identified in soybean.

**Methods:**

We utilized Illumina's SBS sequencing technology to generate high-quality small RNA (sRNA) data from four soybean (*Glycine max*) tissues, including root, seed, flower, and nodules, to expand the collection of currently known soybean miRNAs. We developed a bioinformatics pipeline using in-house scripts and publicly available structure prediction tools to differentiate the authentic mature miRNA sequences from other sRNAs and short RNA fragments represented in the public sequencing data.

**Results:**

The combined sequencing and bioinformatics analyses identified 129 miRNAs based on hairpin secondary structure features in the predicted precursors. Out of these, 42 miRNAs matched known miRNAs in soybean or other species, while 87 novel miRNAs were identified. We also predicted the putative target genes of all identified miRNAs with computational methods and verified the predicted cleavage sites *in vivo *for a subset of these targets using the 5' RACE method. Finally, we also studied the relationship between the abundance of miRNA and that of the respective target genes by comparison to Solexa cDNA sequencing data.

**Conclusion:**

Our study significantly increased the number of miRNAs known to be expressed in soybean. The bioinformatics analysis provided insight on regulation patterns between the miRNAs and their predicted target genes expression. We also deposited the data in a soybean genome browser based on the UCSC Genome Browser architecture. Using the browser, we annotated the soybean data with miRNA sequences from four tissues and cDNA sequencing data. Overlaying these two datasets in the browser allows researchers to analyze the miRNA expression levels relative to that of the associated target genes. The browser can be accessed at http://digbio.missouri.edu/soybean_mirna/.

## Background

Many classes of 18-30 nt small non-coding RNAs (sRNAs) can be characterized based on their functions in gene regulation and epigenetic control in plants, animals and fungi [1,2].

Identification of the complete set of miRNAs and other small regulatory RNAs in organisms is essential with regard to our understanding of genome organization, genome biology, and evolution [3]. There are three important classes of endogenous small RNAs in plants, animal or fungi: micro RNAs (miRNAs), short interfering RNAs (siRNAs) and piwi-interacting RNAs (piRNAs). In plants, there are no known piRNA.

MicroRNAs (miRNAs) are small 18-24 nucleotide regulatory RNAs that play very important roles in post-transcriptional gene regulation by directing degradation of mRNAs or facilitating repression of targeted gene translation [4,5]. While siRNA are processed from longer double stranded RNA molecules and represent both strands of the RNA, miRNAs originate from hairpin precursors formed from one RNA strand [6,7]. The hairpin precursors (pre-miRNA) are typically around ~60-70 bp in animals, but somewhat larger, ~90-140 bp in plants. In plants, helped by RNA polymerase II, miRNA gene is first transcribed into pri-miRNA. The pri-miRNAs are cleaved to miRNA precursors (pre-miRNA), which form a characteristic hairpin structure, catalyzed by Dicer-like enzyme (DCL1) [7,8]. The pre-miRNA is further cleaved to a miRNA duplex (miRNA:miRNA*), a short double-stranded RNA (dsRNA) [9]. The dsRNA is then exported to cytoplasm by exportin-5. Helped by AGO1, single-strand mature miRNA will form a RNA-protein complex, named RNA-induced silencing complex (RISC), which negatively regulates gene expression by inhibiting gene translation or degrading mRNAs by perfect or near-perfect complement to target mRNAs [10,11].

Although some soybean miRNA were previously identified [12], the number was small and, therefore, the identification of all soybean miRNAs is far from complete. The aim of this study is to expand the collection of miRNAs expressed in soybean by using a deep sequencing approach with the Illumina Solexa platform. Towards this, we generated Solexa cDNA sequencing data for root, nodule and flower tissues since they are all relevant soybean organs to various studies in legume biology and due to their impact on soybean yield. One of the legume-specific traits is the symbiosis existing between the legume root and soil bacteria leading to the nodule. We think the small RNA content of soybean nodules needs to be established since research in other legume species showed a role for small RNA in nodule development [13,14]. Root tissue is another important organ to analyze due to its role in nutrient-water absorption, which is clearly important to soybean yield. Finally, we selected flower for its direct impact on soybean seed yield. We constructed the small RNA libraries, prepared from these four different soybean tissues and each library was sequenced individually, generating a total of over one million sequences per library. We developed a bioinformatics pipeline using in-house developed scripts and other publicly available RNA structure prediction tools to differentiate the authentic mature miRNA sequences from other small RNAs and short RNA fragments represented in the sequencing data. We also conducted a detailed analysis of predicted miRNA target genes and correlated the miRNA expression data to that of the corresponding target genes using Solexa cDNA sequencing data.

## Methods

### Sources of sequences and assemblies

Illumina's SBS sequencing technology was utilized to generate high-quality reads from four different soybean tissues, including root, seed, flower, and nodule. The Gmax1.01 release version genomic sequences and gene model predictions of Williams 82 soybean genome were acquired from Phytozome [15] and used as a reference genome.

### Small RNA library construction and SBS sequencing

Soybean (Glycine max L. Merr.) cultivar Williams 82 were planted in 5 L pots containing a mixture of two parts Metro-Mix 360 (Scotts-Sierra Horticultural Products Co., Marysville, Ohio) and 1 part vermiculite (Strong-Lite Medium Vermiculite, Sun Gro Horticulture Co, Seneca Illinois). Plants were grown under controlled environmental conditions in a greenhouse, with a temperature regime of 22 ± 3°C/day and 20 ± 3°C/night, and relative humidity ranging from 45% to 65% throughout the day/night cycle. Sunlight was supplemented with metal halide lamps, set to a 15-h day, 9-h night cycle (lights on at 700 h). The soil mixture was kept moist by an automated drip irrigation system, which delivered nutrient solution twice a day (younger plants) or three times a day (older plants). The nutrient solution contained the following concentrations of mineral salts: 1 mM KNO_3_, 0.4 mM Ca(NO_3_)^2^, 0.1 mM MgSO_4_, 0.15 mM KH_2_PO_4 _and 25 μM CaCl_2_, 25 μM H_3_BO_3_, 2 μm MnSO_4_, 2 μM ZnSO_4_, 0.5 μM CuSO_4_, 0.5 μM H2MoO_4_, 0.1 μM NiSO_4_, 1 μM Fe(III)-/N/,/N'-/ethylenebis [2-(2-hydroxyphenyl)-glycine]. Flowers were collected at -2 to 2 days after anthesis. Seeds were harvest at 20 days post anthesis. Root tissues were collected from 3 weeks-old soybean seedlings, which were grown with nitrogen and 1/2 lullien [16] solution in cassions. To collect nodules, soybean plants were grown in aeropontic growth champers for 7 days in 1/2 × Lullien medium [16], with no nitrogen and inoculated with *Bradyrhizobium japonicum *USDA110 to induce nodule formation. Nodules were collected 7, 14, and 21 days after inoculation and pooled for RNA isolation. Total RNA of different tissues were isolated by TRIzol reagents (Invitrogen). Small RNA libraries were constructed and sequenced as previously described [17].

### Bioinformatics analyses of sequencing data

The sequencing reads were generated for all four libraries along with base quality score information. The sequences were initially trimmed for adapter sequences with an in-house trimming procedure and raw abundance read counts were calculated for all unique sequences for each library individually. This final set of reads and corresponding read counts for each library were all combined to generate a list of unique sequences, which are referred to as sequence tags henceforth. These sequence tags were then mapped to the soybean genome assembly using the stand-alone version of Megablast software [18], and the corresponding matching chromosome number, positions and strand were recorded for those mapped to the genome with no mis-matches in sequence tags. The sequence tags not passing this mapping criterion were excluded from further analysis.

We further analysed these 1.2 million, mapped sequence tags against the gene model predictions and identified and updated the information for any sequence tags which overlapped with the coding gene positions on the genome. We also predicted the tRNAs in the soybean genome using tRNAscan-SE version 1.21 software [19] and also looked for sequence similarities using BLAST for the tRNAs in other genomes in the Genomic tRNA database [20]. These searches identified ~1200 tRNA predicted in soybean. The ~2469 sequence tags that mapped to the known tRNAs positions were filtered from further analysis. We also performed rRNA predictions in soybean using RNAmmer version 1.2 [21]. Subsequently, the sequence tags that mapped to the ~620 known rRNAs in soybean were also removed before further analysis. Table 1 shows the number of sequence tags removed due to overlap with the tRNA and rRNA positions per library.

**Table 1:** Statistics of sequenced tags matching genome, tRNA and rRNA

	Root	Nodule	Flower	Seed
**Tags with genome hits**	674226	665947	686029	853162
**Distinct tags with genome hits**	172795	287855	288804	350869
**Tags with genome hits unique to library**	102947	198520	185554	244069
**Tags with tRNA hits**	78680	40622	41114	106561
**Distinct tags with tRNA hits**	756	423	611	719
**Tags with rRNA hits**	2367	2714	1754	1618
**Distinct tags with rRNA hits**	524	644	479	437
**# Sum_use (tags with non tRNA, rRNA genomic hits)**	595546	625325	644915	746601

The raw abundance values for sequence tags in every library were normalized into corresponding transcripts per million (TPM) abundance values using(1)

where n_base is a million (1,000,000); #sum_use is number of tags found to hit genomic positions of non-tRNA and non-rRNA regions. To ensure that we were working with good quality and expressed small RNA sequences, we further removed any sequence tags where the sum of TPM abundance from all the four libraries was <20 TPM [22]. At the conclusion of these filtering steps, 113,399 sequence tags were considered in our further analysis.

### miRNA identification

We extracted 200 bp upstream and downstream genomic sequences for all the sequence tags that passed the >20 TPM abundance filtering criterion and further predicted the hairpin-like RNA secondary structures for all. The secondary structure was predicted by DINAMelt program using default RNA3.0 parameters [23]. To ensure the stem-loop precursor could be precisely processed into mature miRNA, the predicted structures were examined according to the following criteria [8]:

i. The candidate miRNA and miRNA* should come from opposite stem-arms and must be entirely within the arm of the hairpin;

ii. miRNA::miRNA* duplex mismatches were restricted to four or fewer;

iii. The frequency of asymmetric bulges is restricted to less than one and the size should be less than 2 bases.

Following these stringent filtering criteria, we identified 129 putative miRNA sequence tags that were compared against the downloaded miRBase [24], containing known miRNA sequences for soybean and other genomes, using FASTA to identify the already known miRNA and differentiate them from the novel miRNA. We identified a total of 129 putative miRNA sequences, of which 42 matched the already known miRNAs in miRBase while 87 were novel miRNAs.

### miRNA family assignment

The 129 predicted miRNA sequences were further assigned to miRNA families using sequence similarity to other known miRNA (including already known soybean miRNA) in the miRBase database. The putative miRNA sequences, along with the known soybean miRNA sequences, were used for multiple alignments using ClustalW [25] and the miRNA families were assigned based on the dendogram tree. 42 miRNAs were assigned families based on matches to known miRNAs, while the 87 novel miRNAs will get new families using miRBase Registry.

### miRNA target gene prediction and experimental validation

The 129 predicted miRNA sequences were later utilized for miRNA target identification using an in-house plant target prediction program following standard rules of miRNA-mRNA interactions [26,27]. The filtering criteria were: a mismatch is given a score of 1, a wobble (G:U mismatch) is given a score of 0.5, and a bulge is given a score of 2. The final entry score was set to 4. At the same time, positions 10 and 11 of miRNA must perfectly match to its target, and there should be no more than one mismatch in miRNA position 2 to 9. Overall, we identified 603 target genes for 78 of the identified miRNA. Further analysis showed that target genes could be predicted for ~73% of known miRNAs and ~54% of novel miRNAs. No targets could be predicted for the remaining ~26% of known miRNA and ~45% of novel miRNA using the above stringent score cutoff. To validate the miRNA-cleavage site, PolyA RNA was extracted and a 5'-RACE reaction was performed using a Gene Racer core kit (Invitrogen, Carlsbad, CA) following previously published methods [12].

### Data display and data integration

We deposited the data in a soybean genome browser based on the architecture provided by USCS genome browser. The Gmax1.01 release version genomic sequences and gene model predictions were downloaded from Phytozome and formatted to be used as annotations for our soybean genome browser. The mapped chromosome number and position information of the four small RNA library sequence tags was converted into a BED format and uploaded to the browser as user tracks. The final putative miRNA list was also converted to BED format and also color-coded based on expression pattern, as well as miRNA length.

### Solexa transcriptome data analysis and integration

We also generated Solexa cDNA sequencing data for three out of the four soybean tissues (root, nodule and flower) that were used for small RNA library construction. In addition, we also have Solexa cDNA reads derived from mRNA isolated from green pods. The raw sequencing reads for the four tissues were aligned to the soybean reference genome acquired from Phytozome using the MAQ-0.6.6 version software [28]. The aligned read positions were further converted into the WIG format and uploaded to the soybean genome browser as user tracks for the four tissues individually. The transcriptomic data for the libraries were normalized against the total number of soybean reads identified in each tissue and analysed to generate the expression abundance values for all the genes in soybean. The expression values of the miRNA were compared against the miRNA target gene abundance values to provide some valuable insight into the predicted target gene regulation by the respective miRNA. We calculated Spearman correlation coefficients between the two and looked specifically into any miRNA-target gene pairs with a strong negative correlation.

## Results

### Overview of small RNA sequencing results

**Figure 1:**
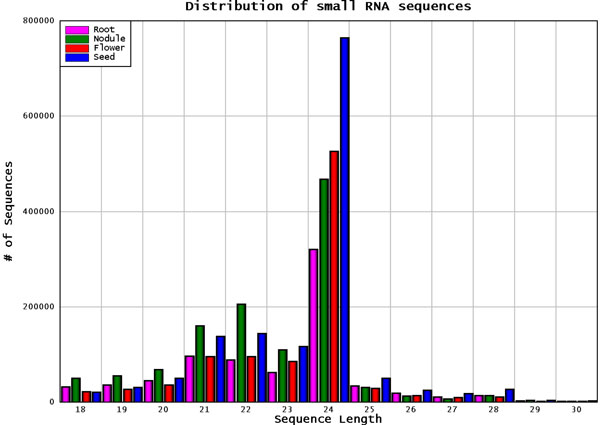
**Distribution of sequence reads in the small RNA libraries**. Distribution of the various lengths of the small RNA sequences in the four libraries examined. The majority of the reads are 21, 22 and 24 nucleotides in length.

The sequencing reads for all four libraries were combined and a unique list of sequence tags was generated with the corresponding raw abundance read counts, for simplicity. Although the total combined sequencing data had over 1 million reads per library, the unique sequence tags were less than that. This highlights the fact that, while there are some common sequence tags being expressed in all or multiple libraries, there are some specifically present only in some libraries. The sequence tags varied in length from 18 bp through 30 bp as seen in Figure 1, with the highest abundance being around the lengths 21, 22 and 24 bp. Some of the variability in small RNA length may be the result of artefacts introduced by the cloning, trimming and/or sequencing techniques. The seed tissue library followed by the flower library had the largest number of sequencing reads and sequence tags with perfect genomic mapping, with 244,069 and 185,554 unique sequence tags in each library, respectively.

### Novel and known miRNA identification

**Figure 2:**
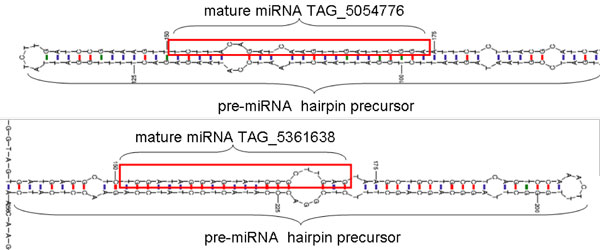
**miRNA hairpin structure**. The hairpin structure of the pre-miRNA precursor predicted using the Quickfold. The mature miRNA sequences TAG_5054776 and TAG_5361638 are highlighted in red, respectively.

**Figure 3:**
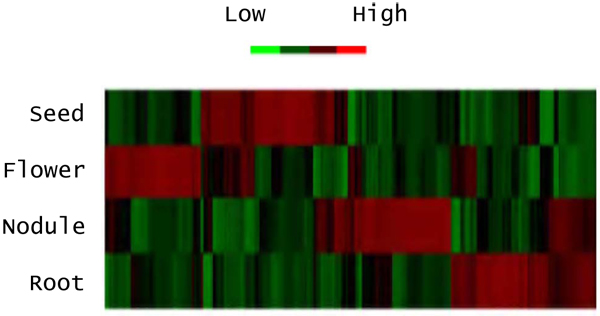
**miRNA expression pattern**. The expression pattern of the129 predicted miRNA across the four small RNA libraries including root, nodule, flower and seed. The expression patterns were clustered together based on the expression pattern across libraries. The low and high expression levels are shown in green and red respectively.

The bioinformatic analyses identified 129 miRNAs based on similarities to conserved known miRNAs, as well as novel miRNAs predicted from their hairpin secondary structure features derived from genomic sequences. The list of the 129 predicted miRNAs can be found in the Additional File 1. Figure 2 shows the hairpin structure of a predicted miRNA. Figure 3 shows the ranked, expression values for the four, different soybean tissues for each of the 129. Based on our filtering criteria, the novel miRNAs identified not only had to have a sum of expression >20 TPM from the 4 small RNA libraries but also could not overlap with the genomic loci of already annotated soybean miRNAs or other classes of non-coding RNAs. The resulting set of sequences and their respective RNA structures were analyzed to distinguish genuine miRNA precursors from other RNAs that contain similar RNA structures. We also observed that the sequenced tags were comprised of both the miRNA sequence as well as the miRNA* sequence, although the miRNA* was certainly less abundant in all four libraries than the miRNA. In total, the miRNA* sequences were identified for ~105 predicted miRNA amongst all the sequenced libraries.

### miRNA target gene prediction

**Figure 4:**
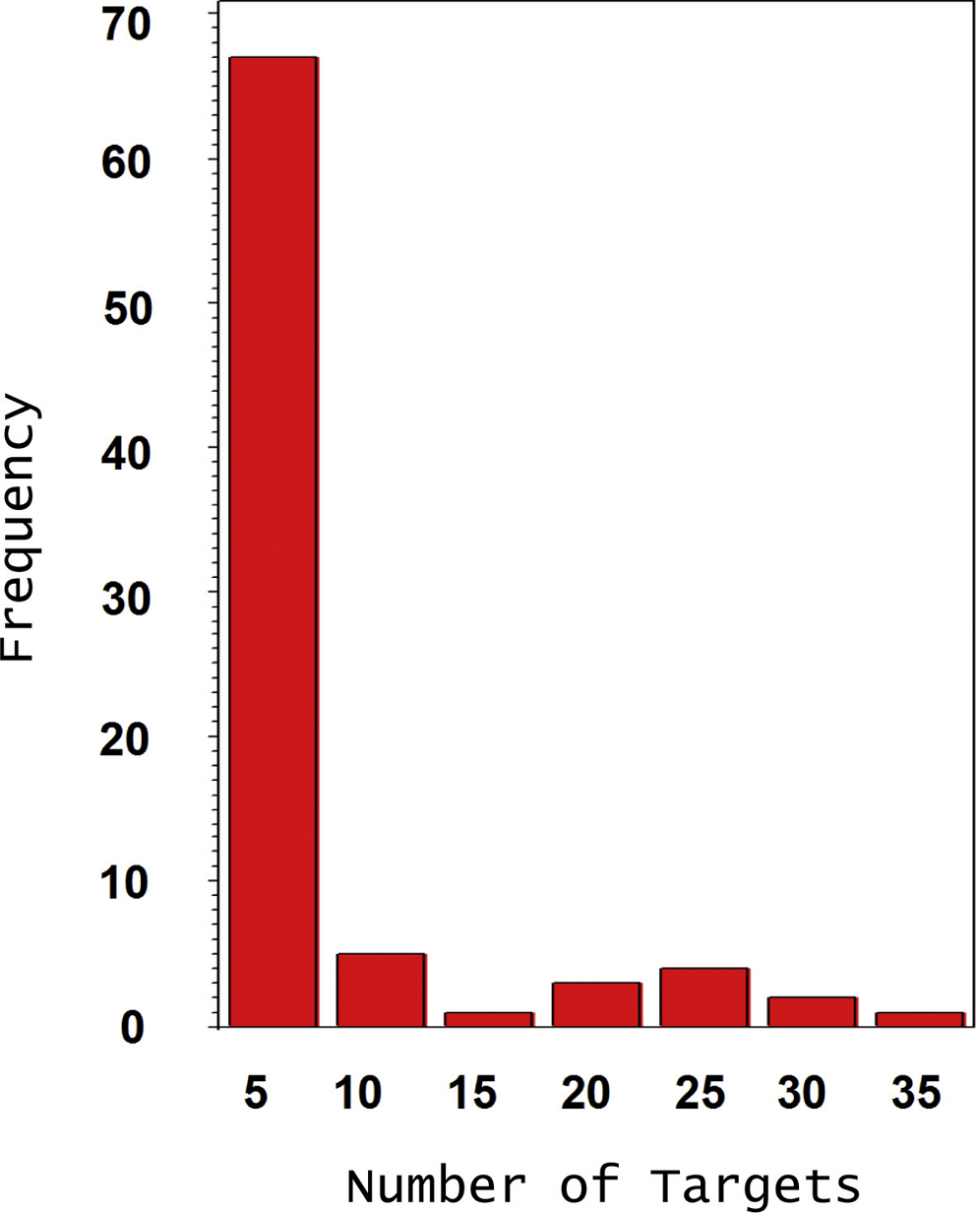
**The frequency distribution of predicted targets for the various miRNAs**. The frequency distribution of the number of target genes predicted per miRNA using the in-house developed plant target prediction program.

**Figure 5:**
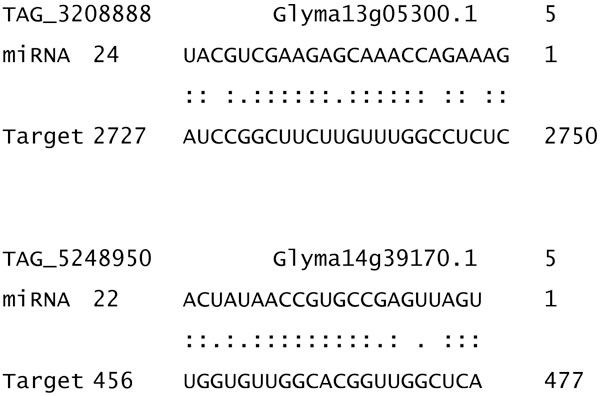
**Sequence alignment between miRNA and its predicted target genes**. The alignment between the miRNA and predicted target gene sequences where ":" indicates a perfectly complementary base and "." indicates a G:U wobble.

**Figure 6:**
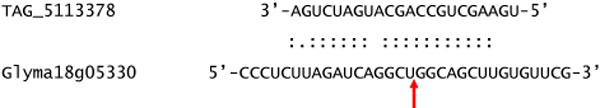
**miRNA target gene cleavage site validated by 5"RACE**. miRNA TAG_5113378 cleavage site on its target gene Glyma18g05330 as identified by 5' RACE is highlighted by red arrow. In the alignment ":" indicates a perfectly complementary base and "." indicates a G:U wobble.

We computationally predicted putative target genes for the 129 identified miRNAs. We identified overall 603 targets from 78 miRNAs, including 174 transcription factors. Among 78 miRNA, 29 miRNAs have one predicted target. TAG_1080847 has the most number with 37 predicted targets, including 12 AP2 domain transcription factors (Figure 4). Figure 5 shows the complementary alignment between the miRNA and the predicted target gene sequences. We selected some miRNAs and conducted 5' RACE experiments to validate the cleavage site and miRNA predicted targets. Figure 6 shows examples of the cleavage site for miRNA TAG_5113378 and its target gene Glyma18g05330 encoding a putative ARF transcription factor.

### Soybean Genome Browser and Solexa transcriptomics data integration

**Figure 7:**
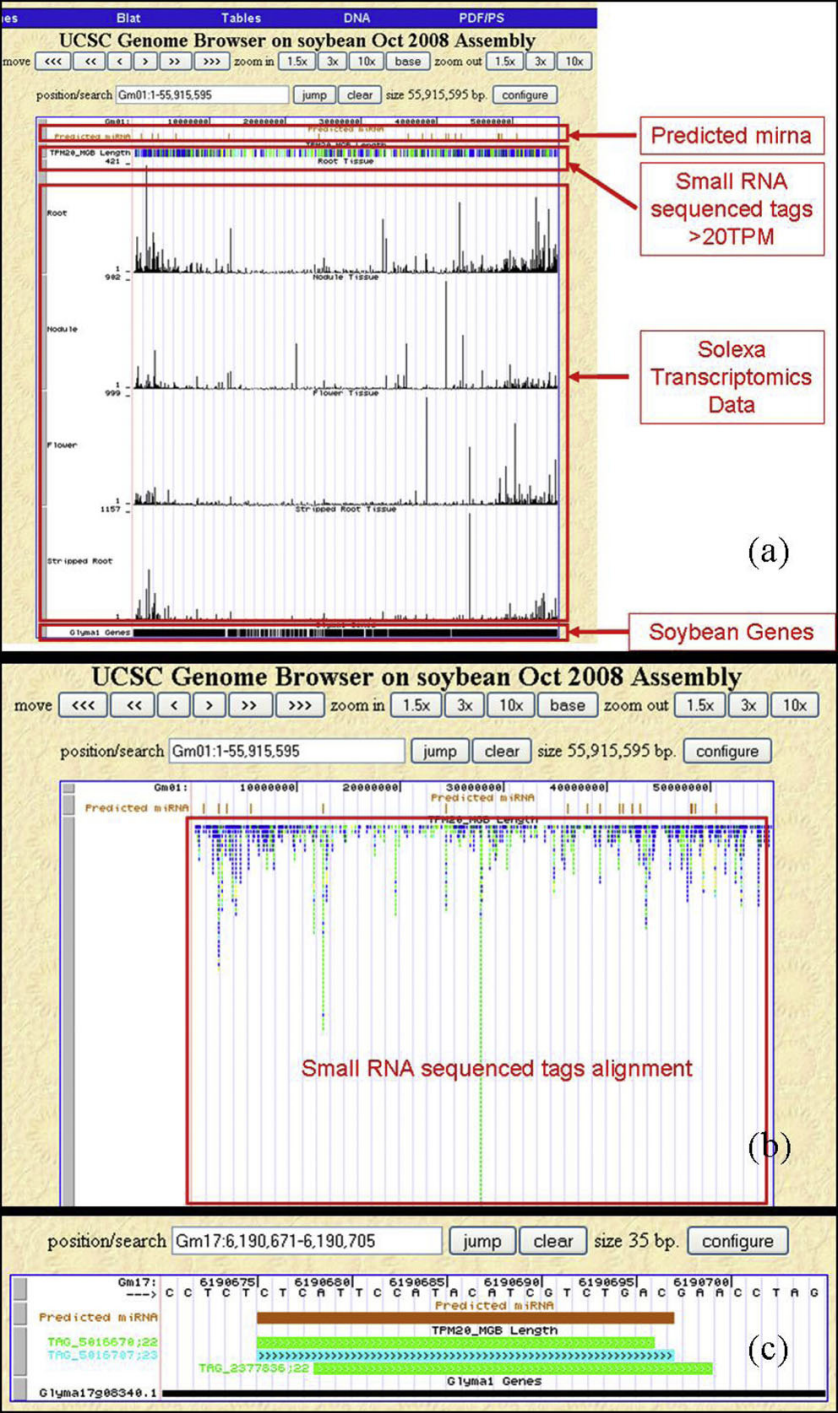
**Soybean Genome Browser**. The soybean genome browser displaying (a) the predicted miRNA sequences, small RNA library sequences >20 TPM and Solexa transcriptomics data from four tissues mapped onto chromosome 1; (b) full display of small RNA library sequences >20 TPM; and (c) TAG_5016707 predicted miRNA and the surrounding other mapped sequences.

A comparison of the small RNA and cDNA expression data within the soybean genome browser enables rapid correlations between specific miRNA and their predicted target genes. Figure 7 shows the miRNA and small RNA library sequence distribution across the entire genome, one entire chromosome at a time. It also allows visualization of the miRNA overlapping against the soybean release version gene model predictions. The Solexa transcriptomics data add another level of information about the expression of the soybean genes in three of the same tissues used for the miRNA analysis. We omitted the comparison between the seed-derived miRNA and the green pod transcriptomic data since these two conditions are likely not comparable.

## Discussion

The soybean genome browser developed utilizing the architecture provided by the UCSC Genome Browser allows for incorporation of the small RNA data, along with soybean transcriptome sequencing data. Overlaying these two datasets along side the soybean gene model predictions facilitates a complete view of the small RNA library distribution along entire chromosomes in one view. It also allows biologists to compare the miRNA expression levels to that of the predicted target genes to get valuable insight into the regulation patterns.

**Figure 8:**
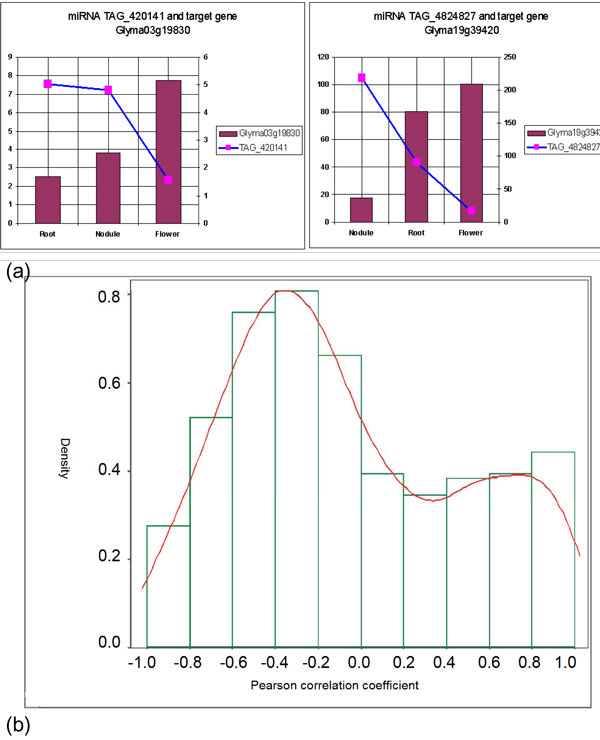
**miRNA and target gene pair regulation**. (a) miRNA and its target gene pair show a strong negative Spearman correlation coefficient between their expression patterns. This strong negative correlation suggests the down regulation of the target genes in response to miRNA expression. (b) The density distribution of correlation coefficient between the miRNA and their target genes expression patterns.

In order to investigate the target gene regulation patterns, we calculated the Spearman correlation coefficients [29] between the miRNA expression levels and the respective target gene expression levels. The miRNAs will degrade their target genes, so miRNA expression should show a negative correlation with the respective target gene. However, some miRNAs could also be co-transcribed with their host genes and targets, especially for miRNAs that are located in intronic regions and self-regulate their host genes. If this is the case, then the expression of some miRNAs will show a positive correlation with their targets [30]. In our results, we observed some strong negative-correlations between miRNAs and their targets (Figure 8a). At the same time, we also observed some positive correlations between the two expression patterns. These results are similar to that reported by Wang for human cancer cells [31]. The density distribution of the correlation coefficients provides a full view of the relationship between miRNAs and their targets (Figure 8b). It shows that there are more cases of negative correlation between miRNAs and their targets than positive correlation. Dugas et al. [32] documented at least one *Arabidopsis *miRNA, miR172, which reduced the accumulation of target protein without significantly effecting target mRNA levels, suggesting that this miRNA may play a role in inhibiting productive translation without affecting mRNA levels. Very little is known about transcriptional and post-transcriptional regulation of miRNAs and, obviously, there is still much to learn. Few studies have sought an understanding of the miRNA complex regulation process in plants and we intend to further expand our observations to studying this relationship by utilizing the unique datasets available to us in soybean.

## Conclusion

Sequencing of four small RNA libraries in soybean which generated over one million sequencing reads per library and its subsequent bioinformatics analyses to identify the authentic known and novel miRNA added 87 new miRNA to the list of known soybean miRNAs. This study encompasses many more soybean tissues than those examined by earlier studies and also provides a unique opportunity to study the relationship between the miRNA expression levels and their regulation of the corresponding target genes utilizing the Solexa cDNA sequencing data derived from the same tissues at the same time. The visualization of the small RNA libraries data alongside the transcriptomics data in the genome browser can help biologists to better understand the dynamics of gene regulation. The many hypothesis generated from this relationship can help advance our understanding of miRNA target gene regulation in the future. As more miRNAs are discovered and their target genes identified, finding biological roles for these interactions enables deeper understanding of gene regulation and of the roles of both the miRNA and its target genes in plant development.

## Competing interests

The authors declare that they have no competing interests.

## Authors' contributions

TJ performed the bioinformatics analysis, developed the in house scripts for the miRNA and the target gene identification, analyzed the Solexa transcriptomics data, deposited the soybean datasets in the genome browser and drafted the initial manuscript. ZY and ML identified target genes, performed hairpin structure prediction and contributed in the discussion of this manuscript. DJ and SP were involved in RNA isolation and library construction. PJG contributed to the experimental design and small RNA libraries. DJS was involved in plant sample production and sequencing of small RNA libraries. AF and GM were involved in Solexa cDNA sequencing. BCM contributed to the experimental design and target prediction. DX provided guidance on the computational analyses. GS conceived the study and obtained funding for the experimental studies. All authors read and approved the final manuscript.

## Supplementary Material

Additional file 1 **Predicted miRNA list identified in soybean**. The list of all 129 predicted miRNA and the miRNA family assigned to those matching already known miRNA. Click here for file

## References

[B1] Brodersen P Voinnet O The diversity of RNA silencing pathways in plants Trends Genet 2006 22 268 280 10.1016/j.tig.2006.03.003 16567016

[B2] Lippman Z Martienssen R The role of RNA interference in heterochromatic silencing Nature 2004 431 364 370 10.1038/nature02875 15372044

[B3] Zhang BH Pan XP Cannon CH Cobb GP Anderson TA Identification and characterization of new plant microRNAs using EST analysis Cell Res 2005 15 336 360 10.1038/sj.cr.7290302 15916721

[B4] Carrington JC Ambros V Role of microRNAs in plant and animal development Science 2003 301 336 338 10.1126/science.1085242 12869753

[B5] Glazov EA Cottee PA Barris WC Moore RJ Dalrymple BP Tizard ML A microRNA catalog of the developing chicken embryo identified by a deep sequencing approach Genome Res 2008 18 957 64 10.1101/gr.074740.107 18469162 PMC2413163

[B6] He L Hannon GJ MicroRNAs: Small RNAs with a big role in gene regulation Nat Rev Genet 2004 5 522 531 10.1038/nrg1379 15211354

[B7] Chapman EJ Carrington JC Specialization and evolution of endogenous small RNA pathways Nat Rev Genet 2007 8 884 896 10.1038/nrg2179 17943195

[B8] Meyers BC Axtell MJ Bartel B Bartel DP Baulcombe D Bowman JL Cao X Carrington JC Chen X Green PJ Criteria for annotation of plant MicroRNAs Plant Cell 2008 20 3186 3190 10.1105/tpc.108.064311 19074682 PMC2630443

[B9] Hannon GJ RNA interference Nature 2002 418 244 251 10.1038/418244a 12110901

[B10] Matzke M Matzke AJM Kooter JM RNA: guiding gene silencing Science 2001 293 1080 1083 10.1126/science.1063051 11498576

[B11] Zhu JK Reconstituting plant miRNA biogenesis PNAS 2008 105 9851 9852 10.1073/pnas.0805207105 18632572 PMC2481364

[B12] Subramanian S Fu Y Sunkar R Barbazuk WB Zhu JK Yu O Novel and nodulation-regulated microRNAs in soybean roots BMC Genomics 2008 9 160 174 10.1186/1471-2164-9-160 18402695 PMC2335117

[B13] Boualem A Laporte P Jovanovic M Laffont C Plet J Combier JP Niebel A Crespi M Frugier F MicroRNA166 controls root and nodule development in Medicago truncatula Plant J 2008 54 876 887 10.1111/j.1365-313X.2008.03448.x 18298674

[B14] Combier JP Frugier F de Billy F Boualem A El-Yahyaoui F Moreau S Vernie T Ott T Gamas P Crespi M MtHAP2-1 is a key transcriptional regulator of symbiotic nodule development regulated by microRNA169 in Medicago truncatula Genes Dev 2006 20 3084 3088 10.1101/gad.402806 17114582 PMC1635144

[B15] Phytozome http://www.phytozome.net/soybean

[B16] Lullien V Barker DG de Lajudie P Huguet T Plant gene expression in effective and ineffective root nodules of alfalfa (Medicago sativa) Plant Mol Biol 1987 9 469 478 10.1007/BF00015878 24277133

[B17] Nobuta K Lu C Shrivastava R Pillay M De Paoli E Accerbi M Arteaga-Vazquez M Sidorenko L Jeong DH Yen Y Distinct size distribution of endogenous siRNAs in maize: Evidence from deep sequencing in the mop1-1 mutant PNAS 2008 105 14958 14963 10.1073/pnas.0808066105 18815367 PMC2567475

[B18] Zhang Z Schwartz S Wagner L Miller W A greedy algorithm for aligning DNA sequences J Comput Biol 2000 7 203 14 10.1089/10665270050081478 10890397

[B19] Lowe TM Eddy SR tRNAscan-SE: A program for improved detection of transfer RNA genes in genomic sequence Nucl Acids Res 1997 25 955 964 10.1093/nar/25.5.955 9023104 PMC146525

[B20] Chan PP Lowe TM GtRNAdb: A database of transfer RNA genes detected in genomic sequence Nucl Acids Res 2008 37 Database D93 D97 18984615 10.1093/nar/gkn787 PMC2686519

[B21] Lagesen K Hallin PF Rodland E Staerfeldt HH Rognes T Ussery DW RNammer: consistent annotation of rRNA genes in genomic sequences Nucleic Acids Res 2007 35 3100 8 10.1093/nar/gkm160 17452365 PMC1888812

[B22] Meyers BC Tej SS Vu TH Haudenschild CD Agrawal V Edberg SB Ghazal H Decola S The use of MPSS for whole-genome transcriptional analysis in Arabidopsis Genome Res 2004 14 1641 1653 10.1101/gr.2275604 15289482 PMC509274

[B23] Markham NR Zuker M DINAMelt web server for nucleic acid melting prediction Nucleic Acids Res 2005 33 W577 W581 10.1093/nar/gki591 15980540 PMC1160267

[B24] Griffiths-Jones S Saini HK van Dongen S Enright AJ miRBase: tools for microRNA genomics Nucleic Acids Res 2008 36 Database D154 D158 17991681 10.1093/nar/gkm952 PMC2238936

[B25] Higgins D Thompson J Gibson T Thompson JD Higgins DG Gibson TJ CLUSTAL W: improving the sensitivity of progressive multiple sequence alignment through sequence weighting, position-specific gap penalties and weight matrix choice Nucleic Acids Research 1994 22 4673 4680 10.1093/nar/22.22.4673 7984417 PMC308517

[B26] Allen E Xie Z Gustafson AM Carrington JC microRNA-directed phasing during transacting siRNA biogenesis in plants Cell 2005 121 207 221 10.1016/j.cell.2005.04.004 15851028

[B27] Schwab R Palatnik JF Riester M Schommer C Schmid M Weigel D Specific effects of microRNAs on the plant transcriptome Developmental cell 2005 8 517 27 10.1016/j.devcel.2005.01.018 15809034

[B28] Maq: Mapping and Assembly with Qualities http://maq.sourceforge.net/index.shtml

[B29] Hogg RV Craig AT Introduction to Mathematical Statistics 1995 5 New York: Macmillan 338 400

[B30] Baskerville S Bartel DP Microarray profiling of microRNAs reveals frequent coexpression with neighboring miRNAs and host genes RNA 2005 11 241 247 10.1261/rna.7240905 15701730 PMC1370713

[B31] Wang YP Li KB Correlation of expression profiles between microRNAs and mRNA targets using NCI-60 data BMC Genomics 2009 10 218 231 10.1186/1471-2164-10-218 19435500 PMC2686738

[B32] Dugas DV Bartel B MicroRNA regulation of gene expression in plants Current Opinion in Plant Biology 2004 7 512 520 10.1016/j.pbi.2004.07.011 15337093

