# Supplementary material for: Prediction of novel miRNAs and associated target genes in Glycine max
Source: BMC Bioinformatics. 2010 Jan 18;11(Suppl 1):S14. doi: 10.1186/1471-2105-11-S1-S14 (PMC3009485; doi:10.1186/1471-2105-11-S1-S14)
Supplement: Additional file 1 — Predicted miRNA list identified in soybean. The list of all 129 predicted miRNA and the miRNA family assigned to those matching already known miRNA. [file 1471-2105-11-S1-S14-S1.doc]

### Additional file 2 – Predicted miRNA list identified in soybean

The list of all predicted miRNA in soybean.

| **miRNA** | **Sequence** | **Length** | **miRNA Family** |
| --- | --- | --- | --- |
| TAG_1080847 | AGAATCTTGATGATGCTGCA | 20 | miR172 |
| TAG_1086843 | AGAATTTGTGGGAATGGGCTGA | 22 |  |
| TAG_1156939 | AGATATTGGTGCGGTTCAATC | 21 |  |
| TAG_1196776 | AGCCAAGGATGACTTGCCGG | 20 | miR169 |
| TAG_1228200 | AGCGTGATGACGTGACACTCCGTC | 24 |  |
| TAG_1236661 | AGCTCTGTTGGCTACACTTT | 20 |  |
| TAG_1238927 | AGCTGCTGACTCGTTGGCTC | 20 |  |
| TAG_1239028 | AGCTGCTTAGCTATGGATCCC | 21 |  |
| TAG_1289431 | AGGCAGGGCTAGTGACTG | 18 |  |
| TAG_1313632 | AGGGATAGGTAAAACAATGACTGC | 24 |  |
| TAG_1348858 | AGGTGGGCATACTGTCAACT | 20 | miR-764 |
| TAG_1368708 | AGTAGACTCGTCCGATTTTGCGTA | 24 |  |
| TAG_1424694 | AGTGGCGTAGATCCCCACAAC | 21 |  |
| TAG_1539852 | ATAGGACTGTCTTAGAATGGTGTA | 24 |  |
| TAG_175411 | AACAAGACGTGATGACGTGACACT | 24 | miR1124 |
| TAG_1826783 | ATGTCACTGATTAGGCATGATGAT | 24 |  |
| TAG_1853964 | ATGTTGTTATTGGATGATGACGGT | 24 |  |
| TAG_1910126 | ATTCGTGGAAGACTGGCGGATCAA | 24 |  |
| TAG_1911586 | ATTCTAAGACGGTTATCTGGGACC | 24 |  |
| TAG_1925000 | ATTGACCAATCAGAACATGACACA | 24 |  |
| TAG_1933636 | ATTGATTCTGAGAGAACCGGTGTA | 24 |  |
| TAG_1948128 | ATTGGGATTCAGTTGGAGTTGG | 22 |  |
| TAG_1978883 | ATTTCTAGGACATACTACGACGGT | 24 | miR-M95 |
| TAG_206492 | AACCAGGCTCTGATACCATGGT | 22 | miR1511 |
| TAG_2103930 | CAAGTCGTAGCCGGTGTTATTACT | 24 |  |
| TAG_2111568 | CAATAAGAACGTGACATATGACAG | 24 | miR1520 |
| TAG_2117741 | CAATCAGAACATGACACATGACAA | 24 | miR1520 |
| TAG_2117766 | CAATCAGAACATGACACGTGACAA | 24 | miR1520 |
| TAG_2138834 | CAATTGGATCGGTCCAACCGGC | 22 |  |
| TAG_2198226 | CACTGTTGTGCTGGGTGTACCA | 22 |  |
| TAG_2220938 | CAGAGGAAGCAGCACTTGTACC | 22 |  |
| TAG_2250818 | CAGGACTGTCTTAGAAAGCCAGGC | 24 | miR-1739 |
| TAG_2282077 | CAGTCGTGTGATTGTACGGTTCAT | 24 |  |
| TAG_2285480 | CAGTGCATGACTATATCGCCAG | 22 |  |
| TAG_228808 | AACGAAGTGACTCTAACATCGGTT | 24 |  |
| TAG_2292781 | CAGTTGACGTACGTACGGATTGAC | 24 |  |
| TAG_237687 | AACGCGTGATATGTTAACATCGGT | 24 | miR-700 |
| TAG_250440 | AACGTCCAATCAGAACGTGACATG | 24 |  |
| TAG_2514743 | CCGGAAGAGACTTACGGATCAACT | 24 |  |
| TAG_252249 | AACGTGACACGTGACGGTCAACAT | 24 |  |
| TAG_2596033 | CCTTAGGACAGACGTCATGTAG | 22 |  |
| TAG_2696762 | CGATTACCAGAAGGCTTATTAG | 22 |  |
| TAG_2719571 | CGCGAGATCGCACGGAAGAAGGTT | 24 |  |
| TAG_2766096 | CGGATTGTTGATCCGTATGTGCAT | 24 |  |
| TAG_283996 | AAGAAAGCTGTGGGAGAATATGGC | 24 |  |
| TAG_289876 | AAGAACGTGACACATGACAATCAA | 24 | miR1520 |
| TAG_291666 | AAGAACTTCTTCCGCGAGATCGCA | 24 |  |
| TAG_2936170 | CTACTTAGTAGAGATTTGTTGG | 22 | miR-1481 |
| TAG_3026261 | CTGAACCCTAGCGAAGTAAATC | 22 |  |
| TAG_307590 | AAGACGGTACTTACCTCAGTAACA | 24 |  |
| TAG_313456 | AAGAGAATTGTAAGTCACTG | 20 |  |
| TAG_3208888 | GAAAGACCAAACGAGAAGCTGCAT | 24 | miR-49 |
| TAG_3371919 | GACGTGACAGACGGAATATCACAT | 24 |  |
| TAG_353548 | AAGCTCAGGAGGGATAGCAC | 20 | miR390 |
| TAG_357044 | AAGCTTCTTACGGATCAAGTTGAT | 24 |  |
| TAG_3661 | AAAAAACTTACGGATCAAGTTGAT | 24 | miR-1192 |
| TAG_366516 | AAGGACGGTACTTACGTAAGCAAC | 24 |  |
| TAG_3842725 | GGATCAAGCTGATCCGGAAGTGGA | 24 |  |
| TAG_397090 | AAGGTGTGATGGCATGACACTCTG | 24 |  |
| TAG_403165 | AAGTAGACATTCTAAGACGTTGCT | 24 |  |
| TAG_420141 | AAGTGATGACATGACAAGCGAAGT | 24 |  |
| TAG_420348 | AAGTGATGACGTGGTAGACGGAGT | 24 | miR-1224 |
| TAG_4327962 | TAAAATCGTGACATGTGACGGTCA | 24 | miR-1631 |
| TAG_4328849 | TAAAATGTAGACATTCTAAGACGG | 24 | miR851 |
| TAG_433723 | AAGTTGACGTACGTACGGATTGAC | 24 |  |
| TAG_4352519 | TAACAACAGCGGAAGAACCTTCTT | 24 |  |
| TAG_4352630 | TAACAACATTGGATGAGGGTTGGA | 24 | miR-1770 |
| TAG_4353664 | TAACAAGTGGGTTTGTTGACTG | 22 |  |
| TAG_435376 | AAGTTGGCTTATGACATACTA | 21 |  |
| TAG_436432 | AAGTTGTGATGAGAATCAATG | 21 |  |
| TAG_4382278 | TAAGACGGAACTTACAAAGATT | 22 |  |
| TAG_4382558 | TAAGACGGTCGTGATGTCAGCA | 22 |  |
| TAG_4459815 | TACCACTAGTGGTCGCGCCTGGCA | 24 |  |
| TAG_4474154 | TACGCAGGAGAGATGACGCTGT | 22 | miR391 |
| TAG_4483101 | TACGTCATCGCTGAATGGAAGACG | 24 | miR-1418 |
| TAG_4500775 | TACTTTCAAAGACGTTGTTGAG | 22 |  |
| TAG_4507540 | TAGAACTGTCTTAGAATGTGCTAC | 24 |  |
| TAG_4529086 | TAGAGTGTATACTGTGAGAGGCCT | 24 |  |
| TAG_4668181 | TATGGGGGGATTGGGAAGGAAT | 22 |  |
| TAG_4669390 | TATGGTCATACGGATTGTTGAT | 22 |  |
| TAG_4673892 | TATGTGACGGTAAACGGTGACAAG | 24 |  |
| TAG_4675772 | TATGTTAACTGATTTCATGGAT | 22 |  |
| TAG_4676672 | TATGTTGATCCGTATGAGTCGTAC | 24 |  |
| TAG_4691924 | TATTGGATCTCAGTTGAACCGGTC | 24 |  |
| TAG_4692308 | TATTGGCTAGAGATAAGACAAAGA | 24 |  |
| TAG_470709 | AATCAAGGAAATCACGGTCGCG | 22 | miR1509 |
| TAG_470717 | AATCAAGGAAATCACGGTTG | 20 | miR1509 |
| TAG_4714278 | TCAAATGATTTTGTGTCGTTGG | 22 |  |
| TAG_473126 | AATCAGAACATGACACATGACAGT | 24 | miR1520 |
| TAG_473197 | AATCAGAACATGACACGTGATAGT | 24 | miR1520 |
| TAG_473234 | AATCAGAACATGACATGTGACAAT | 24 | miR1520 |
| TAG_473538 | AATCAGACACTGCATTCAAAGACG | 24 |  |
| TAG_4735429 | TCAATCAGAACATGACACGTGACA | 24 | miR1520 |
| TAG_4804419 | TCATCGTCCAATCAGAATGTGACA | 24 |  |
| TAG_482882 | AATCGACTTAGAATGTAGGATGGT | 24 |  |
| TAG_483673 | AATCGATGTAGAAAAGTGATTGGT | 24 |  |
| TAG_4883881 | TCGAAGGTTCTGGAGAGGACTGCA | 24 |  |
| TAG_4917396 | TCGCTTGGTGCAGGTCGGGAAC | 22 | miR168 |
| TAG_493367 | AATCTTAGGGACCAAATTGACAGC | 24 |  |
| TAG_4947764 | TCGGTCGGACCGATCCAATCGGAA | 24 |  |
| TAG_4952164 | TCGTACTCGTCGGGTATCGGGTAT | 24 | miR914 |
| TAG_5026252 | TCTCGGCAAAGAACTAAGAAGAAG | 24 |  |
| TAG_5042008 | TCTGCGAAAATGTGATTTCGGA | 22 | miR-922 |
| TAG_5054776 | TCTTACAGATCAAGTTGATTCGGA | 24 |  |
| TAG_5113378 | TGAAGCTGCCAGCATGATCTGA | 22 | miR167 |
| TAG_5113409 | TGAAGCTGCCAGCATGATCTTA | 22 | miR167 |
| TAG_5176695 | TGAGAAAAGGACGGCAGAAAAGCC | 24 |  |
| TAG_5193213 | TGAGCCAAGGATGACTTGCCGGT | 23 | miR169 |
| TAG_519425 | AATGGACTAAAGAGAAAGGGGCCG | 24 |  |
| TAG_5264650 | TGCAGAGATAGGGACGCGCTTA | 22 |  |
| TAG_5361638 | TGGATAGGAGTATGGGCTTGAG | 22 |  |
| TAG_5473728 | TGTAGTTTCTAAGACGATGCTGAC | 24 |  |
| TAG_5478346 | TGTCAAAGATGTGGCGAATACT | 22 |  |
| TAG_5481286 | TGTCACATCCTGGTTGGACATGAA | 24 | miR-M17 |
| TAG_5483776 | TGTCAGCGGAGTGAGAAGACGAAA | 24 |  |
| TAG_5542829 | TGTGTTGAAAGTTTAACATGACGG | 24 |  |
| TAG_5595238 | TTAACGAAAAAGGACTAACGAC | 22 |  |
| TAG_5693737 | TTATTGTAACTAATTTGTCGGT | 22 |  |
| TAG_5729082 | TTCCACAGCTTTCTTGAACTGT | 22 | miR396 |
| TAG_5757832 | TTCGGAAAAATTCTGGAAGACGTC | 24 |  |
| TAG_5809825 | TTGAAAAGGGACAGCAGAGAAGCC | 24 |  |
| TAG_5828832 | TTGACAGAAGAGAGAGAGCACA | 22 | miR156 |
| TAG_598167 | ACAACGTCTTTGAAAGTAGGCATT | 24 |  |
| TAG_701993 | ACAGAAGATAGAGAGCACAG | 20 | miR156 |
| TAG_744429 | ACATATTATGGGTCTCAGACGGAC | 24 |  |
| TAG_82342 | AAACTTGTAAGATGGTGACATT | 22 |  |
| TAG_890021 | ACGGACACCGAACACGACACGGAC | 24 |  |
| TAG_903560 | ACGGCGTGATATTGGTACGGCTC | 23 |  |
| TAG_910300 | ACGGGTCGCTCTCACCTAGG | 20 |  |
